# Physicochemical Properties, Functionalities, and Antioxidant Activity of Protein Extracts from New Zealand Wild Sea Cucumbers (*Australostichopus mollis*)

**DOI:** 10.3390/foods13172735

**Published:** 2024-08-28

**Authors:** Yuan Wen, Xuan Dong, Leonardo N. Zamora, Andrew G. Jeffs, Siew Young Quek

**Affiliations:** 1 Food Science, School of Chemical Sciences, The University of Auckland, Auckland 1010, New Zealand; ywen426@aucklanduni.ac.nz (Y.W.); xdon261@aucklanduni.ac.nz (X.D.); 2 Cawthron Institute, Private Bag 2, Nelson 7042, New Zealand; leo.zamora@cawthron.org.nz; 3 School of Biological Sciences, The University of Auckland, Auckland 1010, New Zealand; a.jeffs@auckland.ac.nz; 4 Institute of Marine Science, The University of Auckland, Auckland 1010, New Zealand; 5 Riddet Institute, Centre of Research Excellence for Food Research, Palmerston North 4474, New Zealand

**Keywords:** sea cucumber, marine protein, enzyme-assisted extraction, amino acid profile, molecular weight distribution, protein functionality, emulsifying properties, foaming properties, antioxidant activity

## Abstract

This study investigated the physicochemical properties, functionalities, and antioxidant capacities of protein extracts from wild sea cucumber *Australostichopus mollis* collected from four distinct locations in New Zealand. Protein was extracted from sea cucumber body walls using trypsin enzymatic extraction, followed by cold acetone precipitation. The amino acid analysis revealed high glycine (189.08 mg/g), glutamic acid (119.45 mg/g), and aspartic acid (91.91 mg/g) concentrations in all samples. The essential amino acid indexes of the protein extracts (62.96, average) were higher than the WHO/FAO standard references, indicating the excellent protein quality of *A. mollis*. Furthermore, protein extracts from *A. mollis* demonstrated superior emulsifying activity (202.3–349.5 m^2^/g average) compared to commercial soy and whey protein isolates under all tested pH conditions, and enhanced foaming capacity (109.9–126.4%) and stability (52.7–72%) in neutral and acidic conditions. The extracts also exhibited good solubility, exceeding 70% across pH 3–11. Antioxidant capacities (ABTS and DPPH free radical scavenging activity and ferric reducing antioxidant power) were identified in *A. mollis* protein extracts for the first time, with clear variations observed among different locations. These findings elucidate the advantageous functional properties of protein extracts from wild New Zealand *A. mollis* and highlight their potential application as high-quality antioxidant food ingredients.

## 1. Introduction

Sea cucumbers are marine invertebrates found in both deep and shallow oceanic environments and are significant contributors to global marine biodiversity, with over 1500 species identified, of which approximately 100 are recognised for their culinary uses [1]. Wild sea cucumbers are predominantly harvested from the wild and processed and sold as “bêche-de-mer”, “namako”, or “trepang” within the originating fishing nations before being exported to major trade centres for further distribution or direct sale to customers [2]. The high demand for sea cucumbers in international markets has led coastal nations, such as Canada, Turkey, Indonesia, and Pacific Island countries like Fiji, to become prominent exporters [2,3,4].

Sea cucumbers have gained increased attention recently for their impressive nutritional profiles. Prior studies indicate they are abundant in proteins, essential omega-3 and omega-6 fatty acids, and trace elements, making them an excellent food source [2,5]. Additionally, sea cucumbers are substantial sources of collagen, ranging from 30% to 70% of crude protein in their body walls, which has enhanced their appeal in markets focused on marine-derived collagen products [6].

Sea cucumber products are also widely recognised for their excellent natural bioactivity. Commercially available sea cucumbers contain various bioactive compounds with potential health benefits, including wound healing, antioxidant, antimicrobial, anticoagulant, anti-inflammatory, and anticancer effects [7,8,9]. For example, sea cucumber protein hydrolysates from *Apostichopus japonicus* have demonstrated antioxidant and anti-ageing effects in both in vivo and in vitro studies [10]. These findings collectively suggest great potential to increase the added value of harvested sea cucumbers or hydrolysates of their tissues.

Beyond their culinary and medicinal applications, sea cucumber collagen, rich in hydrophilic groups with notable moisture-retention and absorption capacities, has also shown promising applications in cosmetic formulations [11], suggesting its potential use across various industries.

Despite extensive research on commercially popular sea cucumber species, the properties of proteins from New Zealand’s most conspicuous and largest sea cucumber species (*A. mollis*) remain sparsely studied. Therefore, the present study aimed to bridge these gaps by investigating the physicochemical properties, functionality, and antioxidant activity of protein extracts from sea cucumbers collected from the wild. This investigation aimed to deepen the fundamental understanding of these properties, providing invaluable insights into their potential applications and benefits.

## 2. Materials and Methods

### 2.1. Materials and Chemicals

Fresh wild sea cucumbers, *A. mollis*, were collected during the austral winter from four locations across New Zealand: Mahurangi Harbour (36°29′15.54″ S 174°43′11.22″ E), Cable Bay (41°9′30.65″ S 173°24′53.71″ E), Elaine Bay (41°3′15.72″ S 173°46′10.55″ E), and Schnapper Point (41°12′26.26″ S 173°54′39.05″ E). All locations were at least 70 km apart by sea, with the Mahurangi Harbour location over 1200 km distant from the other locations. Six *A. mollis* specimens exhibiting similar conditions were selected from each of the four locations, with an average length of 13.7 cm and an average weight of 45.7 g (wet weight basis) (Table S1). These specimens were processed further as research samples for the analyses in this study, as this is a typical sample size for studies of this nature [12,13].

All chemicals were of analytical grade. Laemmli sample buffer (65.8 mM Tris-HCl; pH 6.8; 26.3% (*w*/*v*) glycerol; 2.1% SDS; 0.01% bromophenol blue), Coomassie Brilliant Blue R-250 Staining Solution, and Coomassie Brilliant Blue R-250 Destaining Solution were purchased from Bio-Rad Laboratories (Hercules, CA, USA). Unstained Protein Standard, Broad Range (10–200 kDa), was purchased from New England Biolabs (Ipswich, MA, USA). Amino acid standards mixture, sodium dodecyl sulphate (SDS), β-mercaptoethanol, Trolox (6-Hydroxy-2,5,7,8-tetramethylchroman-2-carboxylic acid), 2,2′-azino-bis-3ethylbenzothiazoline-6-sulfonic acid (ABTS), 2,4,6-tri(2-pyridyl)-1,3,5-triazine (TPTZ), sodium acetate, and ferric chloride were obtained from Sigma-Aldrich (Castle Hill, Australia). Commercial soy protein isolate (SPI) and whey protein isolate (WPI), with an average protein content of 88%, were purchased from Bulk Nutrients (Tasmania, Australia). Water used in this study was Type 1 water (18.2 MΩ), obtained from a Milli-Q water purification system (Millipore Corporation, Burlington, MA, USA).

### 2.2. Preparation of Sea Cucumber Protein Extract

Sea cucumbers from each location were gutted, thoroughly washed, and the body walls were sectioned into approximately 1 cm × 1 cm pieces. These pieces were then freeze-dried (FreezeZone 12 Plus, Labconco Corp., Kansas, MO, USA) and ground into a fine powder, which was then pooled by location. Protein extraction was performed using an optimised enzymatic process with trypsin (38 °C, pH 8, 12 h) at a sample-to-enzyme ratio of 100:1 (*w*/*w*). A 10 g homogenised sea cucumber powder sample was suspended in Milli-Q water with pre-adjusted pH and controlled temperature at a sample-to-water ratio of 1:30 (*w*/*w*); 100 mg trypsin was added. The mixture was stirred on a magnetic hot plate at 360 rpm at 38 °C for 12 h. Post-extraction, the mixture was heated at 100 °C for 5 min, then centrifuged at 15,000× *g* for 15 min after being cooled to ambient temperature. Four volumes of cold acetone (−20 °C) were added to the supernatant, followed by vortexing and overnight incubation at −20 °C to precipitate the protein. Protein pellets were collected through centrifugation at 15,000× *g* for 10 min, dried using a gentle nitrogen stream, freeze-dried, and finely ground into a homogenous powder, which was then used for all subsequent analyses. The protein content of the extracts from each location was quantified (Table S2) by Kjeldahl analysis and used as a reference for the more detailed analyses conducted (Mahurangi Harbour: 69.3%; Cable Bay: 79.1%; Elaine Bay: 76.4%; Schnapper Point: 71.3% (dry weight basis)).

### 2.3. Physicochemical Properties of Sea Cucumber Protein Extract

#### 2.3.1. Functional Groups Determination

Fourier transform infrared spectroscopy (FTIR) was used to analyse the functional groups of *A. mollis* protein extracts according to Muhammad et al. [14], with minor modifications. The FTIR measurements were performed using a VERTEX 70 spectrometer (Bruker, Billerica, MA, USA) at room temperature (25 ± 3 °C). Infrared spectra were recorded in the region from 400 cm^−1^ to 4000 cm^−1^ with 64 scans and a resolution of 4 cm^−1^.

#### 2.3.2. Thermal Properties Determination

The thermal properties of the protein extracts were assessed using Differential Scanning Calorimetry (DSC). Measurements were conducted with a differential scanning calorimeter (TA Q1000 DSC; TA Instruments, New Castle, DE, USA), according to the method of Cui et al. [15], with some modifications. Homogenised sea cucumber protein isolates were weighed into aluminium pans and sealed. After 5 min of equilibration to ambient temperature, the samples were heated from 20 °C to 210 °C at a ramp of 2 °C/min. The starting temperature (T_0_), transition temperature (T_d_), and enthalpy (ΔH) were determined from the generated thermograms analysed by TA Universal Analysis 2000 v. 4.5A software.

#### 2.3.3. Molecular Weight Distribution of Sea Cucumber Protein Extracts

Sodium dodecyl sulfate-polyacrylamide gel electrophoresis (SDS-PAGE) was performed to determine the molecular weight distribution of sea cucumber protein extracts. Protein extract samples were dissolved in solubilisation buffer (1%, *w*/*v*, SDS;100 mM Tris-HCl; pH 9.5), then mixed with Laemmli sample buffer in the presence or absence of β-mercaptoethanol. After vortexing, the mixtures were heated at 100 °C for 5 min and then centrifuged at 10,000× *g* for 10 min after cooling. Subsequently, 10 µL of the supernatant from each sample was loaded into wells of a 12% precast gel (Mini-Protean TGX Precast Gel, Bio-Rad Laboratories, Inc., Hercules, CA, USA), and the electrophoresis was performed at 110 V for 70 min. Unstained Protein Standard, Broad Range (10–200 kDa) was utilised as the marker. After electrophoresis, the gels were stained with Coomassie Brilliant Blue R-250 (Bio-Rad, Hercules, CA, USA) for one hour and subsequently destained overnight using Coomassie Brilliant Blue R-250 destaining solution.

#### 2.3.4. Amino Acid (AA) Composition Analysis

The protein extracts were hydrolysed using modified methods from Sun et al. [16] and Zhao et al. [17]. Briefly, 5 mg of protein extract was mixed with 1 mL of 6 M HCl containing 0.1% phenol and heated at 110 ℃ for 24 h. After hydrolysis, the solutions were evaporated at 70 °C under N_2_. The remaining residues were redissolved in 2 mL of 80% acetonitrile (MeCN) (MeCN: Milli-Q; 8:2), and the mixtures were thoroughly vortexed and filtered with a 0.22 µm PTFE filter.

The AA compositions were quantified using a modified method based on Huang et al. [18]. Determinations were carried out with an Agilent 1290 Infinity liquid chromatography (LC) system coupled with an Agilent 6460 Triple Quad mass spectrometer (MS) (Agilent 1290 LC & 6460C triple quad MS; Agilent Technologies, Santa Clara, CA, USA). An Agilent InfinityLab Poroshell 120 HILIC-Z column (2.1 × 100 mm, 2.7 μm) was employed to achieve chromatographic separation. Mobile phases A and B, consisting of 20 mM ammonium formate in Type 1 water and 20 mM ammonium formate in acetonitrile, respectively, were used for gradient elution programmed as follows: from 0 to 11.50 min, the composition linearly decreased from 100% to 70% B; and from 11.50 to 12.00 min, it returned to 100% B. The separation was performed at a flow rate of 0.5 mL/min at 35 °C, with an injection volume of 1 μL.

### 2.4. Functionality of Sea Cucumber Protein Extract

#### 2.4.1. Solubility

The solubility of protein extract from the sea cucumber body wall was determined according to Hefter and Tomkins [19] with some modifications. The protein samples were dissolved in pH-pre-adjusted Milli-Q water, and the pH was immediately corrected to the set value using strong HCl and/or NaOH; the solvent volume added during this adjustment was recorded. The mixture was vortexed for 2 min and shaken at 200 rpm for 1 h. Following this, the solution was centrifuged at 10,000× *g* for 20 min. Known-weight supernatants were transferred into the pre-dried weighted (W_1_) crucibles, then heated overnight at 110 °C, and the final weights (W_2_) were recorded. The relative solubility of the protein extracts was calculated using the following equation:

$$Solubility\;(\%)=\frac{\left(W_{2}-W_{1}\right)}{g\;sample/mL\;water}$$

#### 2.4.2. Foaming Properties

The foaming capacity (FC) and foaming stability (FS) were determined according to previous studies [20,21] with modifications. Measurements were conducted at five different pH levels (3, 5, 7, 9, and 11) at the set solid content of 0.6% (*w*/*w*). For each test, 3 mL of the solution was placed into 15 mL centrifuge tubes and homogenised with the Ultra-Turrax homogeniser basic (T25, IKA-Works; Wilmington, NC, USA) equipped with the S25 KD-18G-ST probe (IKA-Works; Wilmington, NC, USA) at 13,000 rpm for 2 min. Foam volumes were recorded immediately and after 30 min at room temperature (22 °C). The foaming capacity (FC) and foaming stability (FS) were calculated according to the equations below:

$$FC\;(\%)=\frac{V_{1}-V_{0}}{V_{0}}\times 100$$

$$FS\;(\%)=\frac{V_{30}-V_{0}}{V_{0}}\times 100$$

where V_0_ is the initial volume before whipping (mL), V_1_ is the volume right after whipping (mL), and V_30_ is the final volume after standing for 30 min (mL).

#### 2.4.3. Emulsifying Properties

The emulsifying activity index (EAI) and emulsifying stability index (ESI) were determined according to the procedure described in previous studies [22,23] with modifications to accommodate the study sample size. Samples were dispersed in Milli-Q water (0.6% *w*/*w*), with measurements conducted across five different pH levels (3, 5, 7, 9, and 11). A 1.8 mL solution was mixed with 0.6 mL of soybean oil and homogenised to an oil-in-water emulsion at 13,000 rpm for 1 min with the Ultra-Turrax homogeniser. After homogenisation, 15 μL of the emulsion (pipetted from the bottom) was diluted 100-fold with 0.1% sodium dodecyl sulphate (SDS, *w*/*v*) solution at 0 and 10 min. Absorbance readings of these diluted solutions were recorded separately at 500 nm using Ultraviolet-Visible Spectrophotometry (UV-1800, Shimadzu, Kyoto, Japan). EAI and ESI were calculated according to the following equations:

$$EAI\left(m^{2}/g\right)=\frac{2\times 2.303\times A_{0}\times F}{c\times \varphi \times L\times 10^{4}}$$

$$ESI\;(min)=\frac{A_{0}}{A_{0}-A_{10}}\times t$$

where A_0_ and A_10_ are the absorbances of the diluted emulsion at 0 min and 10 min, respectively; F is the dilution factor (100); c is the initial protein concentration of the solution; φ is the volume fraction of oil in the oil-in-water emulsion (25%); L indicates pathlength; and *t* is the time for standing (10 min).

### 2.5. Antioxidant Activity of Sea Cucumber Protein Extract

#### 2.5.1. DPPH Free Radical Scavenging Activity Assay

The DPPH (2,2-diphenyl-1-picrylhydrazyl) assay was conducted with modifications from the method described by Chen et al. [24]. Briefly, 50 µL of protein extract solution (2.5 mg/mL) was loaded into a 96-well plate, followed by 150 µL of DPPH methanolic solution (40 mg/mL, made within 24 h). The mixture was incubated for 60 min at room temperature in the dark. The absorbances were read at 517 nm by the EnSpire^TM^ Multimode Plate Reader (2300, PerkinElmer-Wallac Oy, Turku, Finland), with readings taken thrice. Milli-Q water was used as a blank, and the standard curve was made by Trolox solutions at serial concentrations of 2.5, 5, 10, 25, 50, 100, 150, and 200 µM. The DPPH radical scavenging activities for different protein extracts were expressed as micromoles (μM) Trolox equivalents (TE) per gram (μM Trolox/g) of protein extracts.

#### 2.5.2. ABTS Free Radical Scavenging Activity Assay

The ABTS radical scavenging activity was determined according to the methods described by Han and Zhao [25] with modifications. The ABTS stock solution (7 mM ABTS and 140 mM potassium persulfate) was incubated in the dark for 16 h at room temperature; the ABTS working solution was made by diluting the stock solution with methanol until reaching an absorbance of 0.70 ± 0.02 at 734 nm. Immediately after adding 50 µL of protein extract solutions to the 96-well plate, 150 µL of ABTS working solution was added and then incubated in the dark for 10 min at room temperature. The absorbance at 734 nm was determined by the EnSpire^TM^ Multimode Plate Reader (PerkinElmer, Waltham, MA, USA). The ABTS radical scavenging activities for different protein extracts were expressed as micromoles (μM) Trolox equivalents (TE) per gram (μM Trolox/g) of protein extracts.

#### 2.5.3. Ferric Reducing Antioxidant Power (FRAP) Assay

The FRAP was assayed according to Binsan et al. [26] with modifications. In brief, 50 µL of protein extract solutions were mixed with 150 µL of FRAP reagent (300 mM acetate buffer (at pH 3), 10 mM TPTZ solution, and 20 mM FeCl_3_ (10:1:1, *v*/*v*/*v*)) in 96-well plates and incubated for 30 min at room temperature in the dark. After incubation, the EnSpireTM Multimode Plate Reader measured the absorbances at 593 nm wavelength. Milli-Q water was used as a blank, and the standard curve was made by Trolox solutions at serial concentrations. The FRAP radical scavenging activities for different protein extracts were expressed as micromoles (μM) Trolox equivalents (TE) per gram (μM Trolox/g) of protein extract.

### 2.6. Statistical Analyses

All experiments were conducted at least in triplicate. The equal masses of lyophilised protein extract powder from each sampling location were treated as technical replicates, denoted “n”. One-way variance analysis (ANOVA) was used to compare mean values among sea cucumber source locations and was conducted using IBM SPSS (version 28.0.1.0, IBM Corp., Armonk, NY, USA). Any overall differences among means identified by the ANOVA were isolated using pairwise Tukey’s Honestly Significant Difference (HSD) post hoc tests. All statistical testing used a significance level of 0.05.

## 3. Results and Discussion

### 3.1. Physicochemical Properties of Sea Cucumber Protein Extracts

#### 3.1.1. Functional Groups

**Figure 1:**
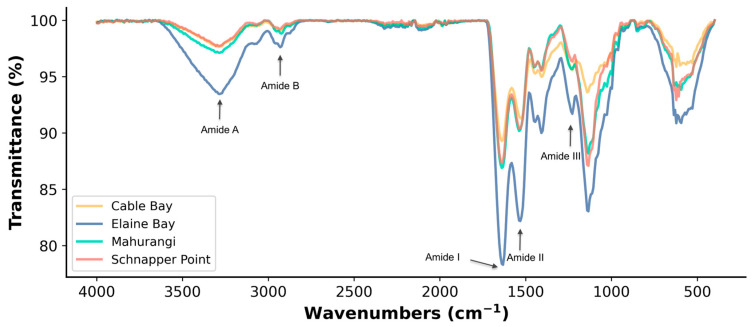
Fourier-transform infrared spectra of protein extract from *A. mollis* from four locations.

The FTIR spectra of protein extracts from *A. mollis* showed characteristic amide frequencies ranging from 400 to 4000 cm^−1^, including amide A, B, amide I, II, and III (Figure 1). Similar functional groups were observed across the different protein extracts, consistent with their protein homology. The functional groups identified in this study are consistent with those reported for other sea cucumber species, such as *Stichopus monotuberculatus* and *A. japonicus* [6,27].

However, slight shifts in the amide bands were noted among sea cucumbers collected from different locations, except for amide III at 1230.47 cm^−1^; details are provided in the Supplementary Materials (Table S3). Amide A peaks were observed in all samples, ranging from 3290.24 cm^−1^ to 3281.60 cm^−1^. Specifically, the lowest wavenumber for amide A (3281.60 cm^−1^) was observed in the protein extract from Mahurangi Harbour. According to Zhong et al. [6], the shift of amide A could be associated with the hydrogen or O-H binding coupled with the N-H group, indicating a higher involvement of N-H groups in hydrogen bonding in the samples from Mahurangi Harbour. Amide B was found in the C-H stretching regions of saturated hydrocarbons, shifting from 2926.73 cm^−1^ (Mahurangi Harbour) to 2932.52 cm^−1^ (Elaine Bay). The shift in amide B to a higher wavenumber is related to increased free -NH^3+^ groups from the N-terminal lysine residue [6]. The highest wavenumber observed in the sample from Elaine Bay, which also had the highest lysine content, indicates a higher presence of free -NH^3+^ groups.

Amides I, II, and III ranges are typical characteristics of proteins directly related to the shape of the peptides in proteins [11]. Amide I raises from the stretching vibration of -C=O associated with the N–H bending vibrations, C-N stretching, and CCN deformation [28], also related to the hydrogen bond coupled with COO- [27]. The wavenumber of amide I in the four samples is relatively similar. Only the sample from Elaine Bay had a slightly higher wavenumber at 1637.41 cm^−1^, while the others were all 1635.48 cm^−1^. The higher amide I wavenumbers suggest a more regular secondary structure within the protein extract, whereas lower wavenumbers indicate that the extract contains a higher proportion of irregular secondary structures, such as random coils, compared to other forms [29,30,31,32]. Amide II showed slight fluctuations, ranging from 1531.33 cm^−1^ in Cable Bay to 1537.12 cm^−1^ in Mahurangi Harbour and Elaine Bay samples. This shift could be related to the formation of hydrogen bonds by N-H groups, as amide II represents the degree to which the N-H group participates in forming hydrogen bonds with adjacent chains [6]. Thus, the higher wavenumber observed in the protein extract from Schnapper Point indicated that samples from this site could have more and/or stronger hydrogen bonds [33] than samples from other sites. The differences observed among samples could be attributed to variations in amino acid profiles, the side chains of different amino acids, and other factors such as the number of polar residues involved in metal chelation and hydrogen bonding [34,35].

#### 3.1.2. Thermal Properties

The thermal properties and thermodynamic parameters are summarised in Table 1. The average thermal denaturation temperature was 45.6 ± 1.5 °C, with enthalpies ranging from 0.32 to 0.71 J/g. The endothermic peaks around 37 °C to 40 °C could mainly be attributed to the denaturation of collagenous tissue proteins, myosin and paramyosin [36]. Comparatively, the denaturing temperatures of *A. mollis* protein extracts observed in this study align closely with the previously reported denaturing temperatures of *A. mollis* (46.7 °C) [37] and were slightly lower than that of *A. japonicus* (52.74 °C) [38]. Notably, similar denaturation temperatures were reported in *A. japonicus*’ digestive tract protein isolate at 46.55 °C, but with a higher enthalpy of 3.99 J/g observed [20]. The denaturation temperatures of protein extracts in this study were also similar to those reported for aquatic proteins, such as those from squid [39] and abalone [36].

The thermal properties of proteins are closely linked to their structural and conformational characteristics. Rao and Caflisch [40] reported that denatured state proteins could exhibit significant heterogeneity, manifesting in high-enthalpy conformations and low-enthalpy traps due to changes in the secondary and tertiary structures. Kaya and Chan [41] also noted that the cooperative interplay between local and nonlocal protein interactions could affect their thermal properties. Thus, the relatively low enthalpy (ΔH) of *A. mollis* observed in this study could be associated with the denaturation resulting from the enzyme-assisted extraction. Specifically, all samples were subjected to heating up to 100 °C to inactivate trypsin. This high-temperature treatment likely caused significant denaturation of the primary protein structures, thereby altering their thermal properties [34,35].

#### 3.1.3. Molecular Weight Distribution

**Figure 2:**
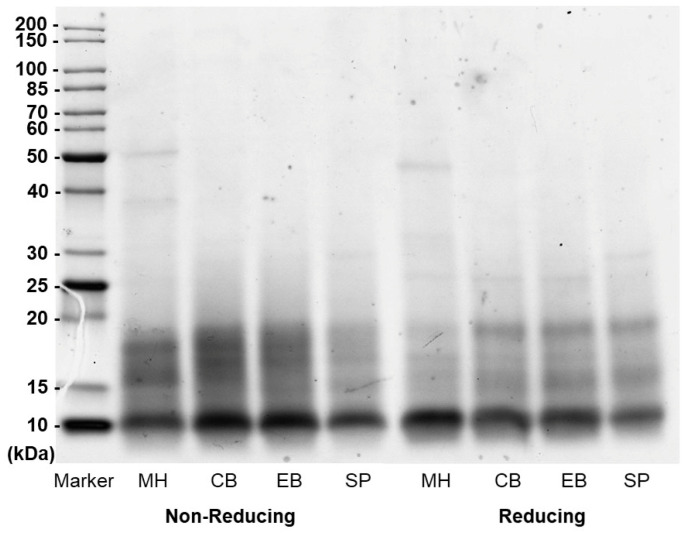
Molecular weight distribution of *A. mollis* protein extract visualised by SDS-PAGE under non-reducing and reducing conditions. MH, Mahurangi Harbour; CB, Cable Bay; SP, Schnapper Point; EB, Elaine Bay.

The molecular weight distribution analysis of protein extracts from *A. mollis* was performed using SDS-PAGE under non-reducing and reducing conditions (Figure 2). Under both conditions, the protein extracts generally exhibited a similar distribution pattern, with a predominant presence of molecules under 20 kDa, especially around 10 kDa. Distinct bands with slightly higher molecular weights were observed in Mahurangi Harbour and Schnapper Point samples. Specifically, a band at approximately 30 kDa was identified only in the Schnapper Point sample, while a band of similar intensity at around 40 kDa and a slightly more intense band at 50 kDa were observed exclusively in the Mahurangi Harbour samples.

The distribution patterns were compared under reducing and non-reducing conditions to understand the impact of disulfide bonding on molecular weights. Under reducing conditions, where disulphide bonds were broken, noticeable shifts in band density were observed. The diffusely distributed bands between 15–20 kDa decreased in density, and the bands clustered around 15 kDa showed increased intensity. Slightly different from the other samples, Mahurangi Harbour samples showed a significant reduction of bands in the 15–20 kDa region and clustering around 10 kDa, which may indicate that proteins in this region of the Mahurangi Harbour samples contain more disulphide bonds. These variations observed between non-reducing and reducing conditions suggest the presence of disulphide bridges in these fractions [42].

Comparatively, the molecular weight distribution pattern of protein extracts of *A. mollis* aligned with those reported in protein isolates from the digestive tract of *A. japonicus* [20]. Although different extraction methods were applied, they also found a diffuse distribution manner with most fractions distributed below a molecular weight of 14.3 kDa. Additionally, a study on trypsin-treated sea cucumber ovum hydrolysates reported that the molecular mass distribution of their hydrolysates was mainly under 5 kDa (85%), consisting predominantly of short-chain peptides of less than eight amino acids [43].

#### 3.1.4. Amino Acid Composition

Amino acids, the essential constituents of proteins, are critical in defining the nutritional value of food. The non-essential amino acids (NEAA) dominated the protein extracts of *A. mollis*, accounting for 72–78% of the total amino acids (Table 2). Specifically, glycine (average, 189.08 mg/g), glutamic acid (119.45 mg/g), aspartic acid (91.91 mg/g), and proline (61.10 mg/g) were the predominant amino acids in the protein extract, making up 35.62% of the total amino acids. The dominant amino acids discovered in this study agreed with a similar study on *Holothuria scabra* [44]. Essential amino acids (EAA) comprised approximately 25% of the protein extract, with threonine, lysine, and leucine being the most prominent. Notably, the total EAA content in *A. mollis* from all the locations in this study surpassed that found in *A. japonicus* (131.60–148.10 mg/g dry weight) [45].

The essential amino acid index (EAAI) was employed to evaluate the quality of the protein extracts. While all samples showed relatively similar EAAI values, with an average of 62.96, the sample from Elaine Bay exhibited a significantly lower EAAI than the other three samples (*p* < 0.05). Subsequently, the EAAI values of all tested samples were compared with the World Health Organization/Food and Agriculture Organization (WHO/FAO) recommendations to assess the nutritional value of the sea cucumber protein extracts. Overall, the EAAI values of *A. mollis* were comparable to the FAO/WHO model (60.3), surpassing both the whole egg model (40.8) [46] and abalone (*Haliotis discus hannai*), which ranges from 47.21 to 51.24 and is recognised for its nutritional richness and high protein quality [47]. The elevated EAAI values of *A. mollis* underscore its superior protein quality and nutritional value.

### 3.2. Functional Properties of Sea Cucumber Protein Extracts

#### 3.2.1. Solubility

**Figure 3:**
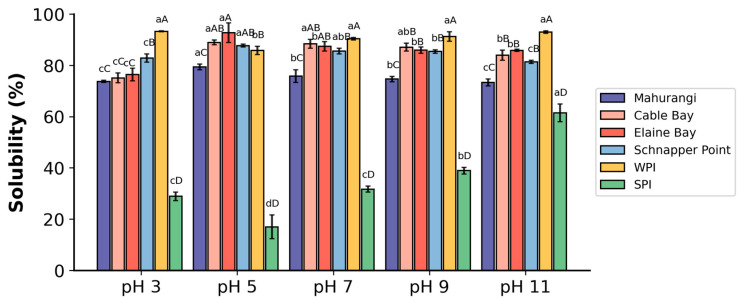
Solubility of A. mollis protein extracts and commercial whey protein isolate (WPI) and soy protein isolate (SPI) at five pH values. Different capital letters indicate a significant difference between different sample sources at the same pH (*p* < 0.05); different lowercase letters indicate a significant difference between the same sample source across different pH values (*p* < 0.05).

Solubility is a crucial property for the potential use of protein extract from *A. mollis* in food and health supplements. The solubility of whole protein extracts from *A. mollis* under five different pH values showed that samples from Cable Bay, Elaine Bay, and Schnapper Point possess similar solubility profiles, with over 80% at pH ranging from 5 to 11 (Figure 3). Conversely, the Mahurangi Harbour samples demonstrated lower solubility across all tested pH levels. The highest solubility for all samples was observed at pH 5, with percentages ranging from 79.38% in Mahurangi Harbour to 92.74% in Elaine Bay samples. At pH lower than 5, the solubility decreased as pH decreased. Although the solubility of *A. mollis* protein extracts was slightly below that of commercial whey protein isolate (WPI), it was significantly better than that of commercial soy protein isolate (SPI), suggesting promising applications in the food industry, particularly under acidic (pH 5) and neutral conditions.

The solubility of protein extracts from *A. mollis* was higher than other sea cucumber protein isolates from *A. japonicus* and *Acaudina leucoprocta* reported in earlier studies [20]. This enhanced solubility in this study could be attributed to the protein extraction and precipitation methods employed. Specifically, this study used trypsin-assisted enzymatic extraction with acetone precipitation to extract proteins from *A. mollis* body walls. According to Liu and Tuo [48], trypsin proteolysis could partially degrade collagen fibrils and release glycosaminoglycan and hydroxyproline by destroying the proteoglycan bridges. They further suggested that trypsin treatment could significantly increase the amounts of total soluble matter (1.44-fold), soluble proteins (4.84-fold), and glycosaminoglycans (2.86-fold), which likely contributes to the observed high solubility in this study. Additionally, enzymatic extraction was reported to reduce the molecular weight of proteins, which can increase the number of free ionisable amino and carboxyl groups and further enhance solubility by promoting protein-water interactions through electrostatic repulsion between peptides [49]. These mechanisms likely contributed to the superior solubilising properties of the proteins extracted from *A. mollis* in this study.

#### 3.2.2. Foaming Properties

**Figure 4:**
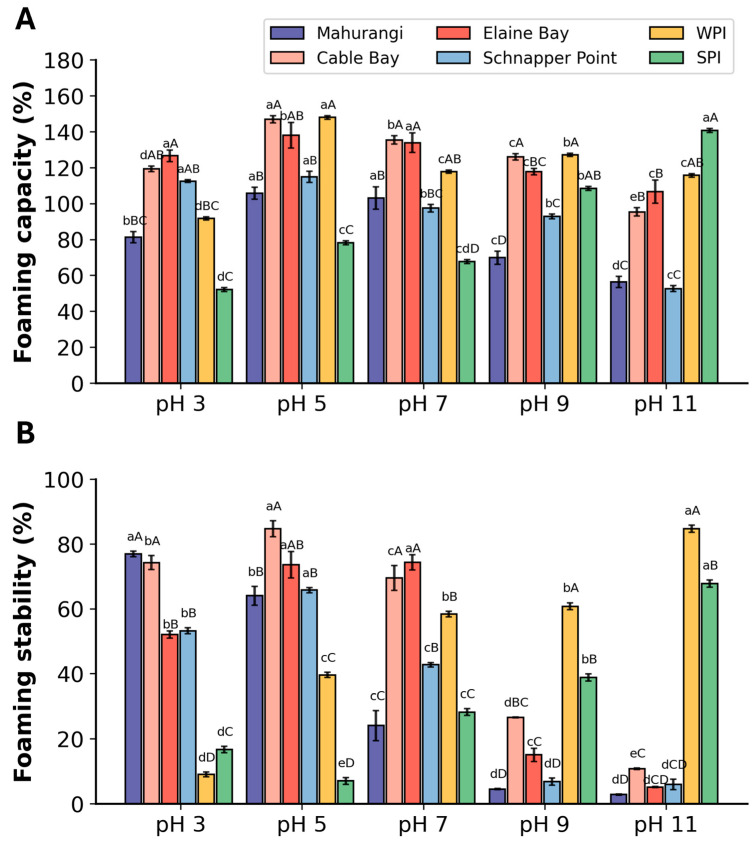
Foaming properties of *A. mollis* protein extracts and commercial whey protein isolate (WPI) and soy protein isolate (SPI) at five pH values. (**A**) foaming capacity; (**B**) foaming stability. Different capital letters indicate a significant difference between different sample sources at the same pH (*p* < 0.05); different lowercase letters indicate significant differences between the same sample source across different pH values (*p* < 0.05).

A comparison of the foaming capacity (FC) of protein extracts across five different pH levels found that the highest foaming capacity occurred at pH 5, where FC of protein extract from *A. mollis* ranged from 105 to 147% (Figure 4A). A clear pH-dependent trend was observed, where FC increased from pH 3 to pH 5, peaking at 5. However, FC gradually decreased beyond this optimum after the pH reached 5. For FS, a distinct pH-dependent trend was also found, with FS increasing at pH 3 to 5 and peaking at pH 5 (Figure 4B). High FS values indicate that the solubilised protein was flexible enough to form a cohesive film to ensure relatively stable foam formation at the water-air interface, thereby maintaining foam stability and preventing subsequent rupture and coalescence [50]. However, significant reductions in FS occurred after pH 5, and poor FS values were observed in all samples under neutral and base conditions.

Among all samples, the protein extracts sourced from Cable Bay and Elaine Bay exhibited superior foaming properties across all pH levels tested. Notably, these extracts also had higher protein content compared to others. Additionally, the foaming properties of *A. mollis* protein extracts showed a similar pH-dependent pattern, aligning with changes in their solubility, which also peaked at pH 5. These findings suggest that the protein content and solubility have positively affected the foaming properties of protein extracts from *A. mollis*. This pattern was also observed in other studies, where high solubility increased protein chain flexibility, facilitating rapid dispersion in water and effective adherence at the water-air interface, demonstrating superior foaming properties [51,52]. Compared to commercial protein isolates, the FC of all extracts was comparable under acidic, neutral, and moderately alkaline conditions but declined significantly above pH 9. In terms of FS, the samples exhibited superior FS under acidic conditions compared to commercial isolates and were also comparable under neutral pH levels but showed much lower FS under alkaline conditions. The differences could be attributed to numerous factors, including differences in particle shape, size and density, different surface characteristics (surface tension, protein adsorption from solution at the liquid/gas interface, surface rheological properties, diffusion of the gas out and into foam cells), and external factors such as evaporation, pressure, and temperature [53]. These findings suggest that *A. mollis* protein extracts are particularly effective for acidic food formulations when maintaining foaming properties is essential.

#### 3.2.3. Emulsifying Properties

**Figure 5:**
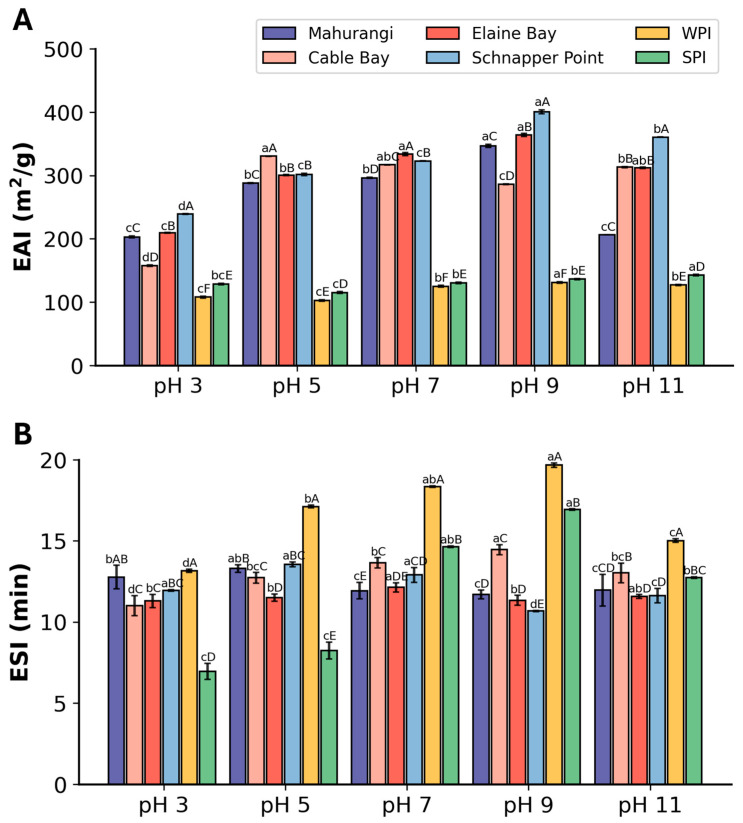
Emulsifying properties of *A. mollis* protein extracts and commercial whey protein isolate (WPI), soy protein isolate (SPI) at five pH values. (**A**) emulsifying activity index; (**B**) emulsifying stability index. Different capital letters indicate a significant difference between the different sample sources at the same pH (*p* < 0.05); different lowercase letters indicate a significant difference between the same sample source across different pH values (*p* < 0.05).

The emulsifying activity index (EAI) of *A. mollis* protein extracts at five different pH levels showed that the protein extracts generally exhibited higher EAI values than commercial ones at all tested pH levels (Figure 5A). Specifically, the extract from Cable Bay showed the highest EAI at pH 5. In contrast, the highest EAI values across the other three protein extracts were observed at pH 9. A clear trend was observed in these three samples, with the EAI increasing from pH 3 to 9 and subsequently decreasing at pH levels above 9. The EAI pattern found in *A. mollis* protein extracts was in line with that found in protein isolate from *A. japonicus*, where the EAI increased with the increase of pH from pH 3 to 8 with a peak EAI of 224.85 m^2^/g [20].

The ESI of the protein extracts from *A. mollis* did not show pH dependency, unlike the pattern found in the EAI of the protein extracts (Figure 5B). The ESI values of different *A. mollis* protein extracts were relatively similar, within the range of 11.01 min to 14.46 min, with an average of 12.25 min. All tested samples showed ESI values comparable to WPI, except under neutral and moderately alkaline conditions, where differences were observed. Under acidic conditions, the samples exhibited significantly better ESI compared to SPI. Compared to the protein isolates of *A. japonicus*, the protein extract from *A. mollis* had similar ESI values under acidic conditions but lower values under alkaline conditions [20].

Numerous factors could influence the emulsifying properties of protein extracts. The type, particle size, and structure of peptides within the protein extracts could affect their emulsifying properties by influencing the conformation at the oil/water interface, surface charge, and interfacial tension [54,55,56]. Furthermore, emulsifier peptides could also influence ESI by providing varying degrees of steric hindrance and electrostatic repulsion, which are critical for emulsion stabilisation at the oil/water interface [57]. Additionally, proteins and peptides with smaller molecular weights tend to diffuse faster at the interfacial surface due to the inverse relationship between protein size and adsorption rate [54,58,59]. The SDS-page results revealed that *A. mollis* protein extracts in this study contained fractions with small molecular weights, likely contributing to the relatively high EAI values and moderate ESI observed. Other influential factors, including the proportion of different secondary structures, the type of enzymes used for extraction, and the degree of hydrolysis, could also contribute to emulsifying properties [60,61]. Consequently, further in-depth and comprehensive research approaches are necessary to understand the emulsifying properties of *A. mollis* protein extracts more thoroughly.

### 3.3. Antioxidant Activity of Protein Extracts from Sea Cucumber Body Wall

**Figure 6:**
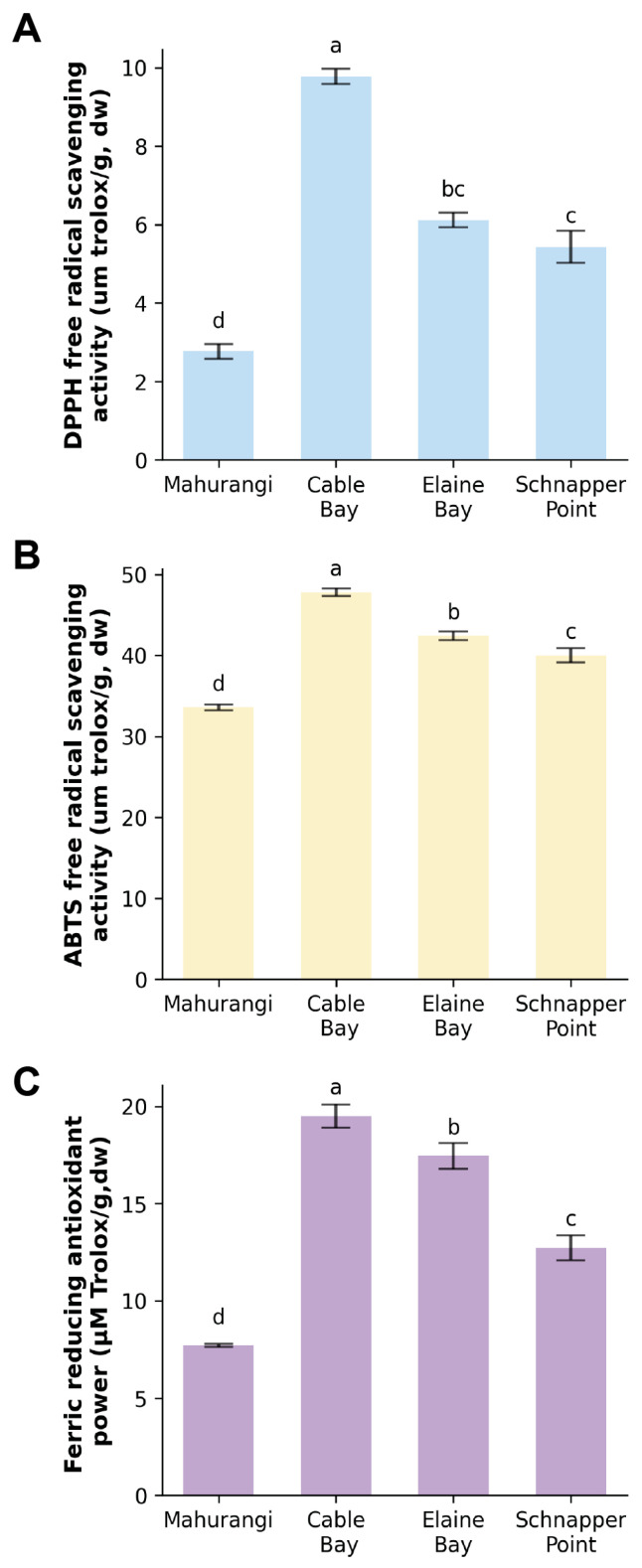
The antioxidant activity of the protein extracts of *A. mollis* ((**A**): DPPH; (**B**): ABTS; (**C**): FRAP). Bars represent the mean with standard deviation. Different letters indicate significant differences between the sampling locations (*p* < 0.05).

The antioxidant properties of *A. mollis* protein extracts were determined using three different assays: DPPH, ABTS, and FRAP, with the most potent antioxidant activity of *A. mollis* protein extract observed in extracts from Cable Bay, giving 9.78 μmol of Trolox/g (DPPH), 20.22 μmol of Trolox/g (FRAP), and 47.81 μmol of Trolox/g (ABTS) (Figure 6). Despite the variations in results obtained by the three assays, a consistent trend was observed. Samples from Elaine Bay and Schnapper Point also exhibited notable antioxidant activity, second to that from Cable Bay, while extracts from Mahurangi Harbour displayed the lowest activity among the tested samples. This trend was also consistent with the protein content in these four samples, indicating that the antioxidant activity of the samples could be proportional to their protein content.

The antioxidant abilities of *A. mollis* protein extracts in this study aligned with those reported in protein hydrolysates from *Cucumaria frondosa* [62]. These findings also corresponded with those of sea cucumber (*A. japonicus*) peptides, which have demonstrated significant antioxidant capacities linked to the presence of low molecular weight peptides [63]. The SDS-PAGE analysis results for *A. mollis* protein extracts strongly confirmed the existence of low molecular weight peptides in the extracts, suggesting that these components may underlie the observed antioxidant activities.

In summary, protein extracts from New Zealand *A. mollis* had considerable antioxidant properties that appear proportional to their protein content. Cable Bay extracts showed the highest antioxidant activity among the four sampling locations. Notably, the antioxidant capacities of *A. mollis* protein extracts were comparable to that of some commonly consumed seafood [54]. Additionally, sea cucumbers contain a wide range of other bioactive compounds, such as holothurian fucoidan, fucosylated chondroitin sulfate, and other polysaccharides with vigorous antioxidant activity [64,65]. While this study focused on protein extracts, future research could explore other bioactive compounds in *A. mollis* from New Zealand.

## 4. Conclusions

The findings demonstrate that the body wall of the New Zealand wild sea cucumber (*A. mollis*) could be a valuable protein resource. All samples shared identical physicochemical properties, including functional groups, thermal properties, molecular weight distribution, and amino acid profiles. Protein extracts displayed complete amino acid profiles, rich in essential amino acids, highlighting their high nutritional quality, as evidenced by their EAAI, revealing their potential use as a protein source. Distinct functional properties were observed in the New Zealand *A. mollis* protein extracts compared to commercial protein isolates. Relatively high solubility across the pH range of 3–11 and higher emulsifying activity of *A. mollis* protein was observed at all pH levels in comparison with commercial protein isolates. Furthermore, the sea cucumber protein extracts showed superior foaming capacity and stability under neutral and acidic conditions compared to commercial protein isolates. Lastly, antioxidant capacity was also observed in all the protein extracts from different locations for the first time. These results suggest significant potential for incorporating New Zealand *A. mollis* protein into the food system, enhancing product characteristics and offering nutritional benefits. The functional properties of sea cucumber protein, assessed for the first time in this study, provide valuable insights for its application in food and supplement products. Further studies are required to illuminate additional aspects, such as spatial and temporal (e.g., seasonal) differences in the biochemical composition of sea cucumbers, the potential for applying different analytical methods and their impact on sea cucumber protein extractions, the long-term stability of protein functionalities and other bioactive substances in New Zealand sea cucumbers.

## Figures and Tables

**Table 1:** Differential scanning calorimetry (DSC) results.

Sample	T_0_ (°C)	T_d_ (°C)	ΔH (J/g Sample)
Mahurangi Harbour	39.33 ± 0.20 ^B^	44.36 ± 1.03 ^B^	0.32 ± 0.05 ^C^
Cable Bay	40.09 ± 0.17 ^A^	47.45 ± 0.56 ^A^	0.43 ± 0.03 ^B^
Schnapper Point	37.79 ± 0.42 ^B^	44.37 ± 1.79 ^B^	0.48 ± 0.01 ^B^
Elaine Bay	40.33 ± 0.32 ^A^	45.41 ± 0.21 ^A^	0.71 ± 0.04 ^A^

Values are presented as mean ± SD (*n* = 5). Values within a column with different uppercase letters differ significantly (*p* < 0.05). T_0_: onset temperature; T_d_: denaturation temperature; ΔH: enthalpy change.

**Table 2:** Amino acid composition (mg/g) of protein extracts of sea cucumber body walls from different locations.

	Amino Acid	Mahurangi Harbour	Cable Bay	Elaine Bay	Schnapper Point
EAA	Threonine	43.89 ± 2.00 ^eB^	50.75 ± 3.16 ^deA^	32.25 ± 3.31 ^fC^	41.2 ± 3.59 ^eB^
	Lysine	38.75 ± 2.91 ^eB^	47.2 ± 4.22 ^eA^	48.03 ± 4.94 ^eA^	33.68 ± 1.10 ^efB^
	Valine	21.08 ± 2.25 ^fgA^	8.83 ± 0.98 ^hijkC^	15.56 ± 0.66 ^ghiB^	22.51 ± 0.78 ^ghA^
	Leucine	25.66 ± 1.72 ^fB^	29.67 ± 1.53 ^fA^	19.88 ± 2.79 ^gC^	27.58 ± 1.05 ^fgAB^
	Isoleucine	15.14 ± 1.35 ^fghA^	16.92 ± 0.40 ^ghiA^	11.97 ± 1.49 ^hijkB^	16.24 ± 0.66 ^hiA^
	Phenylalanine	16.71 ± 0.55 ^fghAB^	18.38 ± 1.63 ^ghA^	18.25 ± 0.24 ^ghA^	15.46 ± 0.68 ^hijB^
	Methionine	6.72 ± 1.15 ^Bij^	7.79 ± 0.21 ^ijkAB^	8.39 ± 0.14 ^ijklA^	7.18 ± 0.78 ^ijklAB^
	Histidine	5.01 ± 0.16 ^Bij^	5.42 ± 0.32 ^jkA^	4.52 ± 0.17 ^klmC^	4.24 ± 0.17 ^jklC^
NEAA	Glycine	172.5 ± 26.6 ^aAB^	202.8 ± 20.0 ^aAB^	168.8 ± 11.3 ^aB^	212.6 ± 24.6 ^aA^
	Glutamic acid	100.4 ± 3.92 ^bC^	121.1 ± 10.8 ^bB^	150.7 ± 10.5 ^bA^	105.7 ± 4.51 ^bC^
	Aspartic acid	87.32 ± 4.04 ^cBC^	92.11 ± 4.96 ^cB^	108.4 ± 5.71 ^cA^	79.85 ± 5.20 ^cC^
	Proline	58.09 ± 3.37 ^dB^	59.86 ± 2.93 ^dB^	65.65 ± 0.44 ^dA^	60.8 ± 0.49 ^dB^
	Arginine	20.69 ± 2.26 ^fgA^	23.55 ± 1.5 ^fgA^	21.18 ± 0.31 ^gA^	20.56 ± 2.08 ^ghA^
	Tyrosine	17.39 ± 3.00 ^fghB^	23.65 ± 1.49 ^fgA^	7.35 ± 0.03 ^jklmD^	12.94 ± 0.76 ^hijkC^
	Alanine	12.39 ± 0.68 ^hijB^	13.56 ± 0.24 ^ghijA^	13.61 ± 0.33 ^ghijA^	13.25 ± 0.43 ^hijA^
	Cysteine	6.26 ± 0.78 ^ijB^	6.71 ± 0.32 ^ijkB^	8.40 ± 0.50 ^ijklA^	4.57 ± 0.24 ^jklC^
	Serine	1.85 ± 0.30 ^jA^	2.14 ± 0.26 ^kA^	2.15 ± 0.24 ^lmA^	1.82 ± 0.26 ^klA^
	Total AA	650.0 ± 38.0 ^C^	730.6 ± 12.0 ^A^	705.2 ± 20.2 ^AB^	679.9 ± 20.7 ^BC^
	Total EAA	173.0 ± 7.38 ^AB^	185.0 ± 10.5 ^A^	158.9 ± 5.25 ^B^	168.1 ± 3.63 ^BC^
	Total NEAA	476.9 ± 31.9 ^B^	545.5 ± 14.6 ^A^	546.2 ± 18.2 ^A^	511.7 ± 22.0 ^AB^
	EAA/NEAA	0.36 ± 0.02 ^A^	0.34 ± 0.03 ^A^	0.29 ± 0.01 ^B^	0.33 ± 0.02 ^A^
	EAAI	65.50 ± 1.82 ^B^	66.65 ± 3.23 ^AB^	57.66 ± 1.51 ^C^	62.04 ± 0.62 ^B^
	Limiting AA	Histidine	Valine	Histidine	Histidine

EAA, NEAA, and EAAI represent essential amino acids, non-essential amino acids, and essential amino acid index, respectively; concentrations are presented as mean ± SD (*n* = 3); values within a row with different superscript capital letters are significantly different between samples from other locations (*p* < 0.05); different superscript letters in lowercase within a column indicate significant differences in contents within samples from the same location.

## Data Availability

The original contributions presented in the study are included in the article and Supplementary Material, further inquiries can be directed to the corresponding author.

## Supplementary Materials

The following supporting information can be downloaded at: https://www.mdpi.com/article/10.3390/foods13172735/s1, Table S1: Characteristics of New Zealand wild *A. mollis* collected from four locations; Table S2: Protein content of the freeze-dried body wall and protein extracts obtained from New Zealand wild *A. mollis* captured from four locations; Table S3: FTIR spectra peaks and frequency assignments of protein extracts from *A. mollis* sampled from four locations.
