# Supplementary material for: Physicochemical Properties, Functionalities, and Antioxidant Activity of Protein Extracts from New Zealand Wild Sea Cucumbers (Australostichopus mollis)
Source: Foods. 2024 Aug 28;13(17):2735. doi: 10.3390/foods13172735 (PMC11395102; doi:10.3390/foods13172735)
Supplement: Supplementary file 1 [file foods-13-02735-s001.zip › foods-3159420-supplementary.pdf]

foods

MDPI

Supplementary Materials

Table S1. Characteristics of New Zealand wild *A. mollis* collected from four locations.

Sampling location	Number of sea cucumbers selected	Weight (average, g)	Body wall (total, g)	Internal organs (total, g)	Length (average, cm)	Date of collection
**Mahurangi Harbour**	6	48.47±3.12	257.5	18.05	13.66±3.15	August 2019
**Cable Bay**	6	46.48±4.35	249.7	15.70	13.71±2.80	
**Schnapper Point**	6	43.51±2.56	246.34	18.71	15.14±3.45	July 2021
**Elaine Bay**	6	44.16±8.63	238.63	19.09	12.34±3.96	

Table S2. Protein content of the freeze-dried body wall and protein extracts obtained from New Zealand wild *A. mollis* captured from four locations.

Sampling location	Protein content (%, dry weight basis)	
	Original body wall	Protein extract
**Mahurangi Harbour**	39.47±0.002^{C}	69.32±0.06^{C}
**Cable Bay**	54.06±0.01^{A}	79.13±0.01^{A}
**Schnapper Point**	45.37±0.002^{B}	76.38±0.17^{B}
**Elaine Bay**	45.50±0.004^{B}	71.25±0.11^{B}

Values are presented as mean ± SD (n=3). Values within a column with different uppercase letters differ significantly ($p < 0.05$).

Table S3. FTIR spectra peaks and frequency assignments of protein extracts from *A. mollis* sampled from four locations.

Common name	Wavenumber (cm^{-1})				Frequency assignment
	Mahurangi Harbour	Cable Bay	Schnapper Point	Elaine Bay	
**Amide A**	3281.60	3290.24	3286.38	3284.46	N-H stretching coupled with O-H stretching
-	2951.80	2951.80	2953.73	2951.80	CH_3 symmetric stretching
**Amide B**	2926.73	2932.52	2930.59	2932.52	CH_2 asymmetric stretching
**Amide I**	1635.48	1635.48	1635.48	1637.41	C=O in-plane stretching/ O-H coupled with COO-
**Amide II**	1537.12	1531.33	1533.26	1537.12	N-H bending coupled with C-N stretching
-	1450.33	1448.40	1448.40	1448.40	CH_3 asymmetric bending
-	1406.97	1408.90	1408.90	1406.97	CH_3 symmetric bending
Amide III	1230.47	1230.47	1230.47	1230.47	PO_2^- asymmetric stretching/ N-H bending coupled with C-N stretching

-: no common names for the spectral region.
